# Coenzyme Q10 and Dementia: A Systematic Review

**DOI:** 10.3390/antiox12020533

**Published:** 2023-02-20

**Authors:** Félix Javier Jiménez-Jiménez, Hortensia Alonso-Navarro, Elena García-Martín, José A. G. Agúndez

**Affiliations:** 1 Section of Neurology, Hospital Universitario del Sureste, Arganda del Rey, Ronda del Sur 10, E-28500 Arganda del Rey, Spain; 2 University Institute of Molecular Pathology Biomarkers, Universidad de Extremadura, E-10071 Cáceres, Spain

**Keywords:** coenzyme Q_10_, tissue concentrations, therapeutics, Alzheimer’s disease, dementia, vascular dementia, Lewy body dementia

## Abstract

It is well known that coenzyme Q_10_ (CoQ_10_) has important antioxidant properties. Because one of the main mechanisms involved in the pathogenesis of Alzheimer’s disease (AD) and other neurodegenerative diseases is oxidative stress, analysis of the concentrations of CoQ_10_ in different tissues of AD patients and with other dementia syndromes and the possible therapeutic role of CoQ_10_ in AD have been addressed in several studies. We performed a systematic review and a meta-analysis of these studies measuring tissue CoQ_10_ levels in patients with dementia and controls which showed that, compared with controls, AD patients had similar serum/plasma CoQ_10_ levels. We also revised the possible therapeutic effects of CoQ_10_ in experimental models of AD and other dementias (which showed important neuroprotective effects of coenzyme Q_10_) and in humans with AD, other dementias, and mild cognitive impairment (with inconclusive results). The potential role of CoQ_10_ treatment in AD and in improving memory in aged rodents shown in experimental models deserves future studies in patients with AD, other causes of dementia, and mild cognitive impairment.

## 1. Introduction

The 1,4-benzoquinone ubiquinone or coenzyme Q_10_ (CoQ_10_), which is present in the majority of tissues in the human body, is an important component of the mitochondrial electron transport, participating in the generation of cellular energy through oxidative phosphorylation, and can be present in tissues in three different redox states: fully reduced (ubiquinol), fully oxidized (ubiquinone), and partially oxidized (semiquinone or ubisemiquinone). In addition to mitochondria, CoQ_10_ is present in peroxisomes, lysosomes, and the Golgi apparatus. CoQ_10_ has important antioxidant properties, with both a direct antioxidant effect of scavenging free radicals, and an indirect one of participating in the regeneration of other antioxidants such as ascorbic acid and alpha-tocopherol, offering protection to cells against oxidative stress processes [1,2]. 

It is well known that one of the most important pathogenetic mechanisms of Alzheimer’s disease (AD) and other neurodegenerative disorders is oxidative stress [3,4,5]. Due to the important antioxidant functions of CoQ_10_, several publications over the last two decades have addressed the issues of both determinations of CoQ_10_ levels in different tissues of patients diagnosed with AD or other types of dementia and on the potential therapeutic role of CoQ_10_ in these diseases (considering experimental studies in animal models of dementia in humans suffering from AD or other dementias). This systematic review and meta-analysis aims to analyze the results of studies addressing the tissular concentrations of CoQ_10_ in patients diagnosed with AD and other dementia syndromes compared with healthy controls, and the results of therapeutic trials of CoQ_10_ in AD (including experimental models of this disease) and in other causes of dementia. 

## 2. Methods

### 2.1. Search Strategy and Criteria for Eligibility of Studies

**Figure 1:**
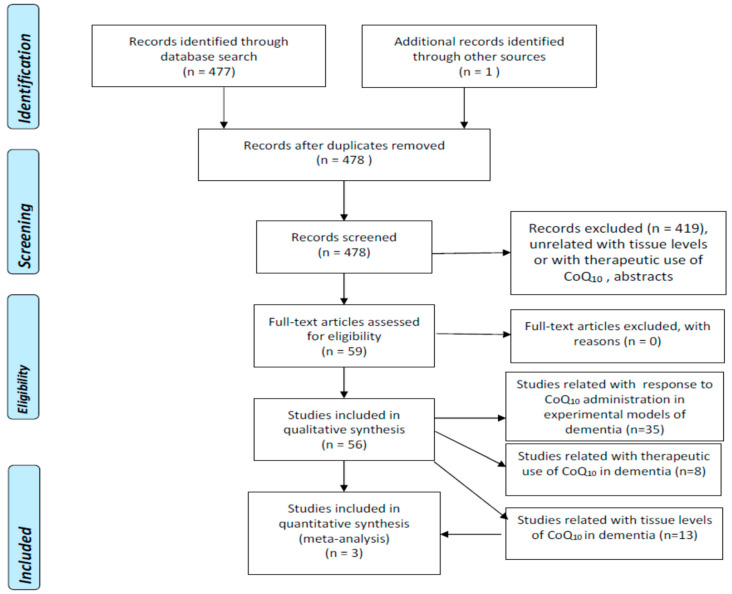
Flowchart for studies assessing tissue concentrations of coenzyme Q10 in dementia (PRISMA) (6, 17).

We undertook a literature search using 3 well-known databases (PubMed, EMBASE, Web of Science-WOS-Main Collection) from 1966 until 31 December 2022. We crossed the term “coenzyme Q_10_” with “Alzheimer’s disease” (188, 403, and 171 items found in PubMed, EMBASE, and WOS, respectively), “dementia” (222, 79, and 86 items found in PubMed, EMBASE, and WOS, respectively), “vascular dementia” (13, 9, and 8 items found in PubMed, EMBASE, and WOS, respectively), “Lewy body dementia” (9, 8, and 11 items found in PubMed, EMBASE, and WOS, respectively) “Lewy body disease” (9, 15, and 25 items found in PubMed, EMBASE, and WOS, respectively), and “mild cognitive impairment” (68, 19, and 34 items found in PubMed, EMBASE, and WOS, respectively). The search retrieved 477 references which were examined one by one by the authors in order to select exclusively those strictly related to the proposed topic. Duplicated articles and abstracts were excluded. We did not apply any language restrictions. Figure 1 represents the flowcharts for the selection of eligible studies which analyzed tissue CoQ_10_ concentrations in patients with different types of dementia, and therapeutic trials with CoQ_10_ in experimental models of dementia or in patients with AD or other dementias according to the PRISMA guidelines [6]. 

### 2.2. Selection of Studies and Methodology for the Meta-Analyses 

We performed a meta-analysis of observational eligible studies assessing the concentrations of CoQ_10_ in tissues of patients diagnosed with AD and/or other causes of dementia and in controls. We extracted the following information: first author, year of publication, country, study design, and quantitative measures. We analyzed the risk for bias with the Newcastle–Ottawa Scale [7]. Table 1 summarizes data from selected studies analyzing tissular concentrations of CoQ_10_ in patients diagnosed with AD, Lewy body dementia (LBD), vascular dementia (VD), and dementia without specification of etiology compared with controls (with the exception of one study that compares the serum/plasma CoQ_10_ of patients with dementia with reference values). 

We converted plasma/serum and CSF CoQ_10_ concentrations to nmol/mL when necessary. The meta-analyses were carried out using the R software package meta [16] and following both the PRISMA [6] (Table S1) and the MOOSE guidelines [17] (Table S2). Because of the high heterogeneity across studies, we applied the random-effects model and used the inverse variance method for the meta-analytical procedure, the DerSimonian–Laird as an estimator for Tau^2^ [18], the Jackson method for the confidence interval of tau^2^ and tau [19], and the Hedges’ g (bias-corrected standardized mean difference) [20]. The statistical power to detect differences in mean values (alpha = 0.05) for the pooled samples was calculated when stated in the text. The meta-analysis was finally only applicable to three studies on serum/plasma CoQ_10_ concentrations in patients with AD compared with controls. 

## 3. Results

### 3.1. Studies Assessing Tissular CoQ_10_ Concentrations 

#### 3.1.1. Alzheimer’s Disease

**Figure 2:**
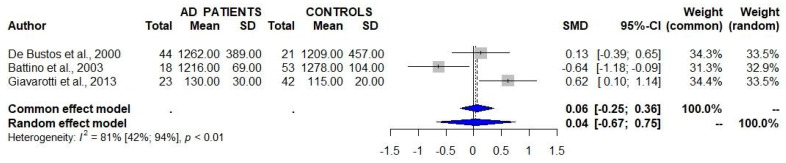
Studies assessing the serum/plasma levels of CoQ_10_ in patients with Alzheimer’s disease (AD) and controls show a lack of significant differences between the two groups. 95% CI 95% confidence intervals; SMD standard mean difference [8,9,10].

A total of three studies that assessed the serum/plasma levels of CoQ_10_ in patients with AD and controls failed to detect significant differences between the two study groups (Table 1, Figure 2) [8,9,10]. One of these studies showed a similar serum/plasma CoQ_10_/cholesterol ratio between AD patients and controls [8].

Isobe et al. [11,12] reported increased total CoQ_10_ and oxidized CoQ_10_ concentrations in the cerebrospinal fluid from AD patients compared with controls, and a negative correlation between oxidized/total coenzyme Q_10_ and duration of the disease.

To date, only two studies have addressed brain CoQ_10_ concentrations in patients with AD. Edlund et al. [21] described the mean values (without SD) of CoQ_10_ in frontal, precentral, temporal, and occipital cortex, and in nucleus caudate, hippocampus, pons, cerebellum, and medulla oblongata of AD patients and controls. They reported a 30–100% increase in CoQ_10_ concentrations in most of these regions; however, the number of AD patients and controls involved in the study was not stated. Kim et al. [22] described a decreased activity of the 25 kDa subunit nicotinamide adenine dinucleotide + hydrogen (NADH):ubiquinone oxidoreductase (complex I) in the temporal and occipital cortex and of the 75 kDa subunit of this enzyme in the parietal cortex of patients with AD compared with controls, but specific measures of CoQ_10_ were not performed. 

Santa-Mara et al. [23] reported the presence of CoQ_10_ in paired helical filaments (aberrant protein aggregates containing tau protein) and in Hirano bodies (neuronal inclusions that are mainly observed in hippocampal neurons and are composed of actin either associated with or not associated with tau) in brain patients with AD, and state that CoQ_10_ was able to induce the formation of aggregates when it was mixed with tau and actin. 

#### 3.1.2. Other Causes of Dementia

Serum CoQ_10_ concentrations and CoQ_10_/cholesterol ratios from patients diagnosed with LBD [13] and VD [8] did not differ significantly from those of controls according to two single studies. 

Yamagishi et al. [14], in a community-based cohort study in Japan involving 6000 Japanese participants aged 40–69 years at baseline, described an inverse association between serum CoQ_10_ concentrations and the risk for disabling dementia, although serum CoQ_10_ levels and serum CoQ_10_/cholesterol ratio did not differ significantly between 65 incident cases and 130 controls. 

Finally, Chang et al. [15] reported “low CoQ_10_ status” in 73% of 80 patients diagnosed with dementia (they used reference values of their laboratory of 0.5-1-7 μM). In addition, they described a correlation between CoQ_10_ status and values of total antioxidant capacity, MiniMental State Examination, amyloid β-42 (Aβ-42), and Aβ-42/40 ratio, but not with tau protein. 

### 3.2. Studies Assessing Therapeutic Response to CoQ_10_ Administration in Experimental Models of AD and Other Dementias

The results of studies assessing the response to the administration of COQ_10_ in different experimental models are summarized in Table 2. 

In general, the administration of CoQ_10_ alone or in combination with other substances (mainly other antioxidants) has been useful to improve the results of clinical tasks related to learning and memory and to improve or prevent oxidative stress, inflammation and cellular death in different models of AD and frontotemporal dementia including aged rodents [24,25,26,27,28,29], aluminium-induced AD in rats [30,31,32], forebrain lesioned rats [33], intracerebroventricular infusion of Aβ-42 [34] or streptozotocin [37,38] or intrahippocampal injection of Aβ-42 [35,36] in rats, transgenic mice with different mutations inducing AD [39,40,41,43,44,45,46] or frontotemporal dementia [42], and cell cultures using different human [25,45,46,48] or rodent cells [49,50,51,52,53,54,55]. On the other hand, Aβ(1-42) decreased CoQ_10_ concentrations in human SH-SY5Y neuroblastoma cells in culture [47].

### 3.3. Studies Assessing Therapeutic Response to CoQ_10_ Administration in Patients with Dementia 

#### 3.3.1. Alzheimer’s Disease 

Table 3 summarizes the results of the eight eligible studies addressing the therapeutic response to CoQ_10_ administration in patients with AD [56,57,58,59,60,61,62,63], although in one of them, an important percentage of patients included were diagnosed with mixed dementia [61]. Two of these studies used an open-label design [56,61] while the others were randomized clinical trials [33].

Imagawa et al. [56], after a preliminary report indicating that therapy with CoQ10, iron, and vitamin B6 was effective as mitochondrial activation therapy in 27 AD patients, reported a significant clinical improvement with this therapy in two genetically confirmed AD patients. Three of the randomized clinical trials showed improvement in neuropsychological assessments in patients treated with CoQ_10_ compared, respectively, with placebo [57,58] or with tacrine [59], while the other two did not show any improvement in comparison with placebo [60,62], although in one of them, the patients treated with CoQ_10_ showed a better outcome than those treated with a combination of α-tocopherol, vitamin C, and α-lipoic acid [62]. 

Karakahya and Özcan [63], in a study using optic coherence tomography (OCT), reported an improvement in retinal ganglion cell loss related to AD with short-term topical administration of CoQ10. Finally, an open-label study showed some degree of improvement in the MMSE score and other neuropsychological tests in patients with AD or mixed dementia [61].

#### 3.3.2. Vascular Dementia (VD)

Kawakami et al. [64] measured CSF levels of homovanillic acid (HVA), 5-hydroxyindole acetic acid (5-HIAA), 3-methoxy-4-hydroxyphenylethylenglycol (MHPG), and noradrenalin (NA) in six patients with cerebrovascular dementia. CoQ_10_ administration during 1–2 months returned to normal CSF levels of HVA, 5-HIAA, and MHPG, which had been previously decreased compared to control values. 

Qi et al. [65], in a randomized clinical trial involving 88 patients diagnosed with VD (44 of them assigned to treatment with butylphthalide plus idebenone as the observational group, and 44 to idebenone as the control group), showed a higher degree of improvement in MMSE, clinical dementia rating scale (CDRS), and ability of daily life (ADL), and a higher decrease in serum IL6, C reactive protein, TNFα, IL1β, CD31+, CDl44+, and endothelin-1 levels in the observational group compared with the control group.

#### 3.3.3. Mild Cognitive Impairment and Normal Aging

García-Carpintero et al. [66], in a 1-year randomized, double-blind, placebo-controlled observational analytical study involving 69 patients diagnosed with mild cognitive impairment (MCI) assigned to CoQ_10_ 200 mg/day (*n* = 33) or placebo (*n* = 36) showed that although CoQ_10_ treatment improved cerebral vasoreactivity (assessed by transcranial Doppler sonography) and inflammatory markers, it did not display any significant improvement in the results of an extensive neuropsychological assessment. 

Finally, Stough et al. [67] designed a 90-day randomized, double-blind, placebo-controlled, parallel group clinical trial involving 104 healthy subjects aged 60 years and over randomized to either CoQ_10_ 200 mg/day or placebo (52 per group), aiming to evaluate the effects of CoQ_10_ in the amelioration of cognitive decline that it should be undergoing. Interestingly, a recent study described a significant association of plasma CoQ_10_ concentrations with cognitive functioning and executive function in elderly people [68].

## 4. Discussion and Conclusions

The possible role of CoQ_10_ in the pathogenesis of AD and other causes of dementia, if any, is far from established with the current evidence. The studies addressing the serum/plasma levels of CoQ_10_, which are scarce and based on a relatively small sample size, were similar for AD patients and controls [8,9,10]. The increased values of total and oxidized CoQ_10_ concentrations in the cerebrospinal fluid from patients [11,12] and in certain brain areas from patients with AD [13], described in single studies, have not had further replication studies and await confirmation. Studies on human AD brain are restricted to a single report of a 30–100% increase in CoQ_10_ concentrations in most of the regions studied (which included the frontal, precentral, temporal and occipital cortex, nucleus caudate, hippocampus, pons, cerebellum, and medulla oblongata) in an unspecified number of AD patients compared with controls [18]. The possibility of induction of aggregates of tau protein and actine by CoQ_10_ and the finding of the presence of this coenzyme in paired helical filaments and Hirano bodies in the hippocampus [23] lends support to the hypothesis of the possible role of CoQ_10_ in AD. Studies reporting on CoQ_10_ concentrations in other causes of dementia are restricted to the measurements in serum/plasma from patients with Lewy body dementia (LBD) [13], vascular dementia [8], and dementia without specification of etiologic diagnosis [14,15].

Because of their antioxidant actions, it was proposed that CoQ_10_ administration could be a potential protective therapy in AD [69,70]. Moreover, an important number of studies have shown a significant neuroprotective and/or clinical effect of the administration of CoQ_10_ in different experimental models of AD, as was previously commented in more detail in the Section 3 [24,25,26,27,28,29,30,31,32,33,34,35,36,37,38,39,40,41,42,43,44,45,46,49,50,51,52,53,54,55] (Table 2). Interestingly, most of the studies performed using cell cultures including human neuroblastoma SH-SY5Y [47] and human MC 65 neuroblastoma cells [25], human umbilical vein endothelial cells [48], rat endothelial [49], cortical [50,51] and brain stem cells [52], hippocampal neurons from fetal mice [53], brain mitochondria isolated from aged diabetic rats [54], and rat pheochromocytoma (PC12) cells [55] have shown a protective effect of CoQ_10_ on the neurotoxic effects of different types of Aβ. In addition, it has been shown that oral administration of CoQ_10_ results in an important increase in serum/plasma CoQ_10_ concentrations in humans [71,72,73] and in rats [73]. 

The potential beneficial effects of CoQ_10_ administration, its good absorption, and the lack of important adverse effects led to some initial short-term randomized clinical trials that showed improvement in several neuropsychological tests in patients with AD treated with CoQ_10_ in comparison with those assigned to placebo [57,58] or the anticholinesterase drug tacrine [59]. However, a further short-term randomized clinical trial failed to determine any benefit except a mild improvement in the ADAS-Cog scores [60]. 

In conclusion, according to the data from the results presented in this review, there are still important knowledge gaps regarding both the suitability of CoQ_10_ as a biomarker of AD and other causes of dementia (studies on this issue in brain, cerebrospinal fluid, and other tissues are scarce) and the possible usefulness of treatment with CoQ_10_ in patients with AD (controversial results of randomized controlled trials with a maximum of 1 year of follow-up) despite the promising neuroprotective effects of CoQ_10_ detected in different models of AD. The design of further studies with a longer-term follow-up period is needed. 

## Figures and Tables

**Table 1:** Coenzyme Q_10_ concentrations in several tissues from dementia patients and healthy controls (HC).

**Alzheimer’s Disease (AD)**							
**Tissue**	**Author, Year [Ref]**	**Parameter**	**AD N**	**AD Mean ± SD**	**HC N**	**HC Mean ± SD**	**Difference in Means (95% C.I.), *p***
**Serum/plasma**	De Bustos et al., 2000 [8]	Total CoQ_10_ (nmol/L)	44	1262 ± 389	21	1209 ± 457	53.00 (−165.26 to 271.26); 0.629
	Battino et al., 2003 [9]	Total CoQ_10_ (nmol/L)	18	1216 ± 69	53	1278 ± 104	−62.00 (−114.55 to −9.45), 0.021
	Giavarotti et al., 2013 [10]	Total CoQ_10_ (nmol/L)	23	130 ± 30	42	115 ± 20	15.00 (2.57 to 27.43), 0.019
	**Total series**	**Total CoQ_10_ (nmol/L)**	**85**	**945.95** ± 573.50	**116**	**844.42** ± 588.71	**Random effects model *p* = 0.911**
	De Bustos et al., 2000 [8]	Total CoQ_10_ / cholesterol (µmol/mmol)	44	0.24 ± 0.05	21	0.23 ± 0.05	0.01 (−0.02 to 0.04), 0.454
**CSF**	Isobe et al., 2009, 2010 [11,12]	Oxidized CoQ_10_ (nmol/L)	30	5.2 ± 1.5	30	1.9 ± 1.3	3.30 (2.69 to 3.91), <0.0001
	Isobe et al., 2009, 2010 [11,12]	Reduced CoQ_10_ (nmol/L)	30	1.4 ± 0.6	30	2.7 ± 0.7	−1.30 (−1.64 to −0.96), <0.0001
	Isobe et al., 2009, 2010 [11,12]	Total CoQ_10_ (nmol/L)	30	6.6 ± 1.2	30	4.6 ± 1.0	2.00 (1.43 to 2.57), <0.0001
	Isobe et al., 2009, 2010 [11,12]	Oxidized/total CoQ_10_	30	0.782 ± 0.188	30	0.413 ± 0.104	0.37 (0.29 to 0.45), <0.0001
**Lewy Body Dementia (LBD)**							
**Tissue**	**Author, Year [Ref]**	**Parameter**	**LBD N**	**LBD Mean ± SD**	**HC N**	**HC Mean ± SD**	**Difference in Means (95% C.I.), *p***
**Serum/plasma**	Molina et al., 2002 [13]	Total CoQ_10_ (nmol/L)	18	960.6 ± 359.1	20	1205.2 ± 362.2	−244.60 (−482.30 to −6.90 to); 0.044
	Molina et al., 2002 [13]	Total CoQ_10_/cholesterol	18	4.67 ± 1.75	20	5.05 ± 1.52	−0.38 (−1.46 to 0.70); 0.478
**Vascular Dementia (VD)**							
**Tissue**	**Author, Year [Ref]**	**Parameter**	**VD N**	**VD Mean ± SD**	**HC N**	**HC Mean ± SD**	**Difference in Means (95% C.I.), *p***
**Serum/plasma**	De Bustos et al., 2000 [8]	Total CoQ_10_ (nmol/L)	17	1130 ± 452	21	1209 ± 457	−79.00 (−379.92 to 221.92), 0.598
	De Bustos et al., 2000 [8]	Total CoQ_10_/cholesterol (µmol/mmol)	44	0.22 ± 0.06	21	0.23 ± 0.05	−0.01 (−0.04 to 0.02), 0.511
**Dementia without Specific Etiologic Diagnosis (DEM)**							
**Tissue**	**Author, Year [Ref]**	**Parameter**	**DEM N**	**DEM Mean ± SD**	**HC N**	**HC Mean ± SD**	**Difference in Means (95% C.I.), *p***
Serum/plasma	Yamagishi et al., 2014 [14]	Total CoQ_10_ (nmol/L)	65	731 ± NA	130	762 ± NA	*p* = 0.32 (according to the authors, SD not provided)
Serum/plasma	Chang et al., 2022 [15]	Total CoQ_10_ (nmol/L)	80	410 ± 21	NA	NA	73% of patients showed low CoQ_10_ status using as reference values 500–1700 nmol/L
Serum/plasma	Yamagishi et al., 2014 [14]	Total CoQ_10_/cholesterol (µmol /mmol)	65	0.14 ± NA	130	0.15 ± NA	*p* = 0.15 (according to the authors, SD not provided)
Serum/plasma	Chang et al., 2022 [15]	Total CoQ_10_/cholesterol (µmol/mmol)	80	0.09 ± 0.04	NA	NA	73% of patients showed low CoQ_10_ status using as reference values 500–1700 nmol/L

**Table 2:** Studies on the effects of coenzyme Q_10_ in different experimental models of Alzheimer’s disease.

Experimental Model	Author, Year [Ref]	Main Findings
AGED RATS	McDonald et al., 2005 [24]	Coadministration of CoQ_10_ and alpha-tocopherol (but not administration of each of these compounds alone) improved learning and memory tasks (assessed by a test that required the mice to rapidly identify and remember the correct arm of a T-maze, and to respond preemptively in order to avoid an electric shock).
AGED MICE	Wadsworth et al., 2008 [25]	Administration of CoQ_10_ decreased protein carbonyls in the brain but had no effect on lipid peroxidation, brain ATP levels, and mitochondrial membrane potential.
	Sumien et al., 2009 [26]	Intake of a low-CoQ_10_ diet did not change age-associated decrements in muscle strength, balance, coordinated running, or learning/memory, whereas intake of CoQ_10_ at a higher amount increased spontaneous activity, worsened age-related losses in acuity to auditory and shock stimuli, and impaired spatial learning/memory of old mice.
	Shetty et al., 2013 [27]	Intake of a low-CoQ_10_ diet did not change age-associated decrements in tests for spatial learning (Morris water maze), spontaneous locomotor activity, motor coordination, and startle reflex. However, intake of high-CoQ_10_ improved spatial learning and decreased protein oxidative damage in the heart, liver, skeletal muscle, and to a lesser extent, in the brain mitochondria.
	Shetty et al., 2014 [28]	Administration of α-tocopherol or α-tocopherol + CoQ_10_ diets improved coordinated running performance. The α -tocopherol + CoQ_10_ diet improved performance in a discriminated avoidance task (α-tocopherol and CoQ_10_ diets alone improved this task to a lesser degree). Both α-tocopherol and CoQ_10_ diets decreased protein damage, this effect being more marked with the α-tocopherol + CoQ_10_ combination.
HYPERCHOLESTE-ROLEMIA-INDUCED AD IN RATS	Ibrahim Fouad, 2020 [29]	Treatment with omega-3 and CoQ_10_ alone or in combination decreased markers of brain oxidative stress and inflammation and serum Aβ levels, regulated cholinergic functioning, and enhanced the functional outcome.
ALUMINIUM-INDUCED AD IN RATS	Ali et al., 2019 [30]	Treatment with CoQ_10_ in combination with vinpocetine partially reversed the changes induced by aluminium chloride (AlCl3) by decreasing malonyl-dialdehyde (MDA), increasing superoxide dismutase (SOD) and total antioxidant total capacity, decreasing IL1β, TNFα, chitinase, β-secretase, Aβ, tau protein, acetyl-cholinesterase, increasing catecholamine and brain-derived neurotrophic factor (BDNF) levels in brain tissue.
	Attia et al., 2020 [31]	Treatment with CoQ_10_ alone or in combination with biotin attenuated the changes induced by AlCl3 (impaired memory, a significant increase in Aβ, lipid peroxides, inflammatory markers—TNFα, IL6, IL1, nuclear factor κB-, caspase-3, and pSer-IRS-1, significant reduction in the antioxidants reduced glutathione and SOD-, pTyr-IRS-1, and p-Akt, reflecting Aβ-induced inflammation and defective insulin signaling, focal aggregations of inflammatory cells and neuronal degeneration).
	Ali et al., 2022 [32]	Treatment with CoQ_10_ reversed changes induced by aluminium by decreasing Aβ and acetylcholinesterase expression, increasing monoamine levels, restoring levels of total antioxidant capacity and superoxide-dismutase, and decreasing MDA, TNFα, and IL6.
FOREBRAIN LESIONED RATS	Nitta et al., 1994 [33]	Administration of CoQ_10_ to forebrain lesioned rats caused an increase in nerve growth factor (NGF) protein and mRNA and in choline acetyltransferase activity, and improved memory tasks such as behavioral deficits in habituation, water maze, and passive avoidance tasks in these animals.
INTRACEREBRO-VENTRICULAR INFUSION OF Aβ(1-42) IN RATS	Yamada et al., 1999 [34]	Coadministration of CoQ_10_ prevented some learning and memory deficits (Y-maze and water maze, but not passive avoidance tasks) in this model without affecting lipid peroxide levels in the hippocampus and cerebral cortex.
INTRAHIPPO- CAMPAL INJECTION OF Aβ(1-42) IN RATS	Singh et al., 2015 [35]	Treatment with CoQ_10_ and minocycline alone improved cognitive performance (reduced transfer latency and increased time spent in the target quadrant in the Morris Water Maze), reduced acetyl–cholinesterase activity, decreased oxidative damage (by reducing lipoperoxide and nitrite level and restoring superoxide, catalase, and reduced glutathione levels), decreased TNFα level, and restored mitochondrial respiratory enzyme complex activities and histopathological alterations induced by Aβ(1-42) in a dose-dependent and synergistic manner.
	Komaki et al., 2019 [36]	Treatment with CoQ_10_ reversed the decreased excitatory postsynaptic potential (EPSP) slope and population spike (PS) amplitude in the hippocampal dentate gyrus after induction of long-term potentiation (LTP) induced by injection of Aβ, reversed the decrease in serum MDA levels and total oxidant levels induced by injection of Aβ, and increased total antioxidant capacity levels.
INTRACEREBRO-VENTRICULAR INFUSION OF STREPTOZOTOCIN IN RATS	Ishrat et al., 2006 [37]	Coadministration of CoQ_10_ prevented learning and memory deficits (loss of cognitive performance in Morris water maze and passive avoidance tests), the increase in markers of oxidative damage (thiobarbituric acid reactive substances, reduced glutathione, protein carbonyl, activities of glutathione peroxidase and glutathione reductase), the decline of ATP in the hippocampus and cerebral cortex, the decrease in choline-acetyl-transferase activity and the increase in acetyl-cholinesterase activity induced by this neurotoxin.
	Sheykhhasan et al., 2022 [38]	Administration of CoQ10-loaded exosomes derived from adipose-derived stem cells improved memory impairment (assessed with the Morris water maze and passive avoidance task), increased BDNF expression, and increased cell density and the transcription factor *SOX2* gene expression in comparison with the administration of CoQ_10_ exosomes derived from adipose-derived stem cells alone.
TRANSGENIC MICE: AD PRESENILIN 1 MUTATION L235P	Yang et al., 2008 [39]	CoQ_10_ administration partially attenuated Abeta overproduction and intracellular Aβ deposit, partially decreased MDA increase, and up-regulated the decreased activity of SOD [24].
	Yang et al., 2010 [40]	CoQ_10_ administration reduced the burden of the amyloid plaques (assessed by immunohistochemistry and magnetic resonance imaging)
TRANSGENIC MICE: TG19959 MUTATION	Dumont et al., 2011 [41]	CoQ_10_ administration improved cognitive performance during Morris water maze testing, decreased brain levels of protein carbonyls (a marker of oxidative stress), decreased brain Aβ42 levels and Aβ protein precursor (AβPP), β-carboxyterminal fragments, and decreased plaque area and number in the hippocampus and the overlying cortex (assessed by immunostained with an Aβ42-specific antibody).
TRANSGENIC MICE: P301 TAU MUTATION (FRONTO- TEMPORAL DEMENTIA)	Elipenahli et al., 2012 [42]	CoQ_10_ administration improved survival and behavioral deficits (it increased locomotor activity and anxiety in open field testing), caused a modest reduction in phosphorylated tau, a significant increase in complex I activity and protein levels, and a reduction in lipid peroxidation in the cortex.
DOUBLE TRANSGENIC MICE: MUTATIONS *TGAPESWE* AND *PSEN1DE9*	Muthukumaran et al., 2018 [43]	Administration of ubisol-Q_10_ (a water-soluble form of coenzyme Q_10_) improved long-term memory, preserved working spatial memory, and inhibited Aβ plaque formation in 18-month-old transgenic mice compared to an untreated transgenic group.
TRIPLE TRANSGENIC MICE: MUTATIONS *PS1M146V*, *APPSWE*, AND *TAUP301L*	Sui et al., 2014 [44]	The administration of CoQ_10_ altered changes in the differentially expressed serum proteins in the transgenic compared with wild-type mice by up-regulating 10 proteins and down-regulating another 10 proteins. Among the proteins modulated by CoQ10, clusterin and α-2-macroglobulin were validated via ELISA assay.
CELL CULTURES: HUMAN SKIN FIBROBLASTS FROM PS1 MUTATED FAMILIAL AD	Ma et al., 2014 [45]	CoQ_10_ treatment decreased reactive oxygen species generation, increased population doublings, and postponed stress-induced premature senescence. CoQ_10_ treatment increased proliferating cell nuclear antigen expression, and decreased levels of manganese-SOD (MnSOD), p21, p16Ink4A and cell cycle regulatory protein retinoblastoma (suggesting a resumption of autophagy).
	Vegh et al., 2019 [46]	Administration of ubisol-Q_10_ caused enhanced expression autophagy-related genes such as *beclin-1* (a major autophagy regulator) and *mitogen-activated protein kinase 8* (*MAPK8/JNK1*, a major activator of beclin-1) avoiding resumption of premature senescence. Withdrawal of ubisol-Q10 treatment led to the return of the senescence phenotype in AD fibroblasts.
CELL CULTURES: HUMAN SH-SY5Y NEUROBLASTOMA CELLS	Qi et al., 2005 [47]	Exposure of these cells to Aβ(1-42) caused, among other effects, enhanced lipid peroxidation and protein oxidation and significant reductions in the total contents of phospholipids, ubiquinone-10, and alpha3 and alpha7 subunit proteins of nicotinic acetylcholine receptors.
CELL CULTURES: HUMAN MC65 NEUROBLASTOMA CELLS	Wadsworth et al., 2008 [25]	Administration of CoQ_10_ showed a neuroprotective effect on the neurotoxic effects induced by the Aβ protein precursor C-terminal fragment (APP CTF).
CELL CULTURES: HUMAN UMBILICAL VEIN ENDOTHELIAL CELLS (HUVECS)	Durán-Prado et al., 2014 [48]	CoQ_10_ pretreatment delayed Aβ incorporation into the plasma membrane and mitochondria, reduced the influx of extracellular Ca^2+^ and Ca^2+^ release from mitochondria due to opening the mitochondrial transition pore after Aβ administration, decreasing O_2_^−^ and hydrogen peroxide (H_2_O_2_) levels, prevented Aβ-induced necrosis and apoptosis, and restored the ability to proliferate, migrate and form tube-like structures in vitro.
CELL CULTURES: RAT BRAIN ENDOTHELIAL CELLS	Frontiñán-Rubio et al., 2021 [49]	CoQ_10_ pretreatment protected endothelial brain cells from Aβ(25–35)-induced damage, preventing nicotinamide adenine dinucleotide phosphate (NADPH) oxidase activity and reducing both reactive oxygen species generation and increase in free cytosolic Ca^2+^ induced by Aβ(25–35) (this prevented apoptosis and necrosis).
CELL CULTURES: PRIMARY CULTURED RAT CORTICAL NEURONS	Choi et al., 2012 [50]	CoQ_10_ protected neuronal cells against Aβ(25–35)-induced neurotoxicity in a concentration-dependent manner by increasing the expression levels of proteins related to neuronal cell survival (p85aPI3K, phosphorylated protein kinase B–Akt-, phosphorylated glycogen synthase kinase-3β, and heat shock transcription factor), and decreasing the levels of proteins associated with neuronal death (cytosolic cytochrome c and cleaved caspase-3). This protective effect was blocked by a phosphatidylinositol 3-kinase (PI3K) inhibitor.
	Wang et al., 2020 [51]	CoQ_10_ pretreatment significantly prevented neurons from Aβ-induced collapse of mitochondrial bioenergetics and perturbations of the protein kinase A (PKA)/cAMP response element-binding protein (CREB) signaling.
CELL CULTURES: CULTURED NEURAL STEM CELLS	Choi et al., 2013 [52]	Co-administration of CoQ_10_ restored the Aβ(25–35) oligomer-inhibited proliferation of neural stem cells by increasing the expression levels of proteins related to the PI3K pathway (p85α PI3K, phosphorylated Akt-Ser473-, phosphorylated glycogen synthase kinase-3β-Ser9-, and heat shock transcription factor). This protective effect was blocked by a phosphatidylinositol 3-kinase (PI3K) inhibitor.
CELL CULTURES: PRIMARY CULTURED HIPPOCAMPAL NEURONS FROM FETAL MICE	Yang et al., 2020 [53]	Administration of CoQ_10_ reversed all the effects induced by sevoflurane anesthesia (decrease in ATP and SOD levels, increase in apolipoprotein E (ApoE) mRNA, total ApoE protein, full-length ApoE, and ApoE fragments, increase in phosphorylated tau and neuroinflammatory factor (TNFα, IL6, and IL1β) expression levels.
CELL CULTURES: BRAIN MITOCHON-DRIA ISOLATED FROM AGED DIABETIC RATS	Moreira et al., 2005 [54]	CoQ_10_ treatment attenuated the decrease in oxidative phosphorylation efficiency and avoided the increase in H_2_O_2_ production induced by Aβ1-40.
CELL CULTURES: RAT PHEOCHROMO-CYTOMA (PC12) CELL LINE	Li et al., 2017 [55]	CoQ_10_ treatment suppressed the protein expression of COX-2 and the level of PGE2 in Aβ(25–35)-injured PC12 cells (this effect was correlated with the suppression of NF-κB activation by CoQ_10_, attenuating neuroinflammation).

**Table 3:** Studies describing the effects of COQ_10_ supplementation in patients with AD. AD: Alzheimer’s disease; ADAS: Alzheimer’s Disease Assessment Scale; ADAS-Cog: ADAS cognitive score; ADAS-Noncog: ADAS non-cognitive scores; ADCS-ADL: Alzheimer’s Disease Cooperative Study Activities of Daily Living; ADL: activities of daily living; CGI-I: clinical global impression improvement; CGI-C: clinical global impression change; CMT: Central macular thickness; DAT: dementia of Alzheimer type; DSS: Digit Symbol Substitution test; GCIPL: Ganglion cell-inner plexiform layer; MMSE: MiniMental State Examination; OCT: optic coherence tomography; RNFL: Retinal nerve fiber layer; SCT: Subfoveal choroidal thickness.

Authors, Year [Ref]	Study Setting	Type of Study	Main Findings	Level of Evidence (Quality Score)
Imagawa et al., 1992 [56]	Combined therapy with CoQ10, iron, and vitamin B6 in 27 AD patients.	Open-label study	Treatment was as effective as mitochondrial activation therapy in 27 AD patients. Treatment induced significant clinical improvement in two genetically confirmed AD patients.	II (NA)
Weyer et al., 1997 [57]	Three hundred patients with mild to moderate degree DAT were prescribed idebenone 30 mg t.i.d. (*n* = 100), idebenone 90 t.i.d. (*n* = 100), or placebo (*n* = 100). Evaluation at baseline, 1, 3, and 6 months including a total score of the ADAS-Total, ADAS cognitive (ADAS-Cog) and noncognitive scores (ADAS-Noncog), CGI-I, MMSE, Digit Symbol Substitution test (DSS) and several scales for the assessment of daily activities (the self- and observer-rating scales NAA and NAB of the Nuremberg Age Inventory NAI and Greene’s Assessment).	Multicenter, randomized, double-blind, placebo-controlled, dosage-ranging trial	Idebenone 90 mg t.i.d. improved significantly and was superior to placebo and idebenone 60 mg t.i.d. in ADAS-Total, ADAS-Cog, ADAS-Noncog, and CGI-global improvement. Safety results (adverse events, vital signs, ECG, and clinical laboratory parameters) were similar for the three groups.	I (>50%)
Gutzmann and Hadler D, 1998 [58]	Four hundred and fifty patients with mild to moderate degree DAT were prescribed placebo for 12 months, followed by idebenone 90 mg for another 12 months (*n* = 153) or idebenone 90 mg tid for 24 months (*n* = 148) or 120 mg ti for 24 months (*n* = 149). Evaluation included a total score of the Alzheimer’s Disease Assessment Scale (ADAS-Total), ADAS cognitive (ADAS-Cog) and noncognitive scores (ADAS-Noncog), CGI-Improvement), the SKT neuropsychological test battery, and the Nurses’ Observation Scale for Geriatric Patients (NOSGER-Total and IADL subscale).	Prospective, randomized, double-blind multicentre study in three parallel groups	During the placebo-controlled period, idebenone showed statistically significant dose-dependent improvement in all the efficacy variables. A further improvement of most efficacy variables was determined in the second year in comparison to the results at the 12-month visit, with a clear dose–effect relationship (placebo < idebenone 90 mg < idebenone 120 mg). Safety results (adverse events, vital signs, ECG, and clinical laboratory parameters) were similar for the three groups.	I (>50%)
Gutzmann et al., 2002 [59]	Two hundred and three patients with mild to moderate degree DAT were prescribed idebenone 360 mg/day (*n* = 104) or tacrine up to 160 mg/day (*n* = 99) for 60 weeks. Evaluation included the Efficacy Index Score (EIS, a combination of improvement in cognitive function, activities of daily living, and global function), the ADAS-Cog score, the NOSGER-IADL score, and the CGI-I.	Prospective, randomized, double-blind, parallel-group multicenter study	A total of 28.8% of the patients on idebenone and 9.1% of the patients on tacrine finalized the follow-up. A total of 50% of the patients on idebenone and 39.4% of the patients on tacrine showed an improvement in at least one of the other (secondary) outcome variables. Patients on idebenone showed a higher benefit from treatment than patients on tacrine.	I (>50%)
Thal et al., 2003 [60]	Five hundred and thirty-six patients diagnosed with probable AD aged over 50 with MMSE scores between 12 and 25 were prescribed idebenone 120, 240, or 360 mg, or placebo (*n* = 136, 138, 133, and 126, respectively) during 1 year. Evaluation included ADAS-Cog, CGIC (primary outcome measures), and measurements of ADL, Behavioral Pathology in Alzheimer’s Disease Rating Scale, and MMSE (secondary outcomes).	Multicenter, double-blind, placebo-controlled, randomized trial	The study was completed by 95, 94, 92, and 96 of the patients assigned to idebenone 120, 240, or 360 mg, or placebo, respectively. Primary outcome measures did not differ significantly between the four groups. In an exploratory two-group analysis comparing all three treated groups combined with a placebo, drug-treated patients performed better on the ADAS-Cog, although CGIC scores did not differ significantly.	I (>50%)
Voronkova and Meleshkov, 2009 [61]	Thirty-five patients were diagnosed with AD (*n* = 9), mixed dementia (*n* = 21), or memory impairment not reaching dementia (*n* = 5). Treatment with CoQ_10_ 120 mg/day for 6 months. Assessment with the Luriya method (memory and especially auditory-speech memory), Clinical Dementia Rating scale (CDRS), CGIC, and MMSE.	Open-label study	Improvement in the MMSE score in patients with mild and moderate dementia. Improvement in daily activities in 27% of patients, including improvement in short-term and long-term memory and attention, speech functions, the performance of kinesthetic, spatial, and dynamic praxis tests, visuospatial gnosis, thought, and writing. Improvement on the CGI scale in 37% of patients.	II (NA)
Galasko et al., 2012 [62]	Seventy-eight patients with mild to moderate AD (66 of them provided serial CSF specimens adequate for biochemical analyses). Random assignment to treatment for 16 weeks with 800 IU/d of vitamin E (α-tocopherol) plus 500 mg/d of vitamin C plus 900 mg/d of α-lipoic acid (E/C/ALA); 400 mg of coenzyme Q_10_ 3 times/day; or placebo (26 to each group; 24, 20, and 12, respectively, provided CSF). Evaluation at baseline and 16 weeks of MMSE and ADCS-ADL scale, and CSF biomarkers related to AD.	Monocenter, randomized, placebo-controlled, double-blind clinical trial	Accelerated decline in MMSE scores occurred in the E/C/ALA group. Changes in CSF Aβ42, tau, and P-tau(181) levels did not differ between the three groups. Cerebrospinal fluid F2-isoprostane levels decreased on average by 19% from baseline to week 16 in the E/C/ALA group but were unchanged in the other groups. Drugs used were well tolerated.	I (>50%)
Karakahya and Özcan, 2020 [63]	Sixty-two patients diagnosed with AD (31 randomized to the treatment group and 31 to the observational group), and 31 healthy controls. The treatment group received topical application of CoQ10 on the retina and choroids. Assessment of CMT, RNFL thickness, GCIPL thickness, and SCT with OCT at baseline and after 6 months.	Monocenter, randomized clinical trial	Increased RNFL thickness in all quadrants in the treatment group, but only significant in the temporal sector (inversely correlated with AD duration). Increased GCIPL thickness in the treatment on average and superonasal sector (inversely correlated with AD severity). Increased ganglion cell-inner plexiform layer in the treatment group.	I (>50%)

## Supplementary Materials

The following supporting information can be downloaded at: https://www.mdpi.com/article/10.3390/antiox12020533/s1, Table S1: PRISMA Checklist; Table S2: MOOSE Checklist.
